# OsnR is an autoregulatory negative transcription factor controlling redox-dependent stress responses in *Corynebacterium glutamicum*

**DOI:** 10.1186/s12934-021-01693-1

**Published:** 2021-10-18

**Authors:** Haeri Jeong, Younhee Kim, Heung-Shick Lee

**Affiliations:** 1 Department of Biotechnology and Bioinformatics, Korea University, Sejong, Republic of Korea; 2 Department of Korean Medicine, Semyung University, Jecheon, Chungbuk Republic of Korea

**Keywords:** *Corynebacterium glutamicum*, *osnR*, Oxidative stress, *sigH*

## Abstract

**Background:**

*Corynebacterium glutamicum* is used in the industrial production of amino acids and nucleotides. During the course of fermentation, *C. glutamicum* cells face various stresses and employ multiple regulatory genes to cope with the oxidative stress. The *osnR* gene plays a negative regulatory role in redox-dependent oxidative-stress responses, but the underlying mechanism is not known yet.

**Results:**

Overexpression of the *osnR* gene in *C. glutamicum* affected the expression of genes involved in the mycothiol metabolism. ChIP-seq analysis revealed that OsnR binds to the promoter region of multiple genes, including *osnR* and cg0026, which seems to function in the membrane-associated redox metabolism. Studies on the role of the *osnR* gene involving in vitro assays employing purified OsnR proteins and in vivo physiological analyses have identified that OsnR inhibits the transcription of its own gene. Further, oxidant diamide stimulates OsnR-binding to the promoter region of the *osnR* gene. The genes affected by the overexpression of *osnR* have been found to be under the control of σ^H^. In the *osnR*-overexpressing strain, the transcription of *sigH* is significantly decreased and the stimulation of *sigH* transcription by external stress is lost, suggesting that *osnR* and *sigH* form an intimate regulatory network.

**Conclusions:**

Our study suggests that OsnR not only functions as a transcriptional repressor of its own gene and of those involved in redox-dependent stress responses but also participates in the global transcriptional regulation by controlling the transcription of other master regulators, such as *sigH*.

**Supplementary Information:**

The online version contains supplementary material available at 10.1186/s12934-021-01693-1.

## Background

*Corynebacterium glutamicum* is a Gram-positive microorganism and classified into the order *Actinomycetales*, which also comprises species such as *Mycobacterium* and *Streptomyces* [1]. *C. glutamicum* is predominantly aerobic and commonly used for the industrial production of amino acids and nucleotides [2]. During the course of fermentation, microorganisms encounter various intracellular and extracellular stresses, among which oxidative stress imposes a major challenge to cells [3, 4]. The responses of *C. glutamicum* cells to stress-causing factors have been studied in some detail, and their molecular regulatory mechanisms are now being unveiled [5].

Reactive oxygen species (ROS), such as hydrogen peroxide (H_2_O_2_), are formed during aerobic respiration and can react with major cellular constituents, including DNA, lipids, proteins, iron-sulfur clusters, and the amino acids cysteine and methionine, in various ways, leading to cell damage [6, 7]. *C. glutamicum* cells are equipped with various enzymatic and non-enzymatic measures, such as catalase and mycothiol (MSH), respectively, that can cope with ROS and stress-caused impairments. In *C. glutamicum*, the *katA* gene encodes the H_2_O_2_-detoxifying catalase, and OxyR acts as the main transcriptional repressor of the *katA* gene [8, 9]. Peroxidases, which have higher affinities for H_2_O_2_ than catalase, have also been detected in *C. glutamicum* [10]. Low-molecular-weight thiols such as mycothiol (1-d-*myo*-inosityl-2-[*N*-acetyl-l-cysteinyl]amido-2-deoxy-α-d-glucopyranoside) function in the maintenance of the cellular redox homeostasis [11–13] by cycling between oxidized and reduced forms [14]. Mycothiol disulfide reductase, encoded by the *mtr* gene, catalyzes production of the reduced form of mycothiol at the expense of the reductant NADPH [15]. Mycothiol has also been implicated in the detoxification of toxins and antibiotics [13, 16, 17]. In addition, thioredoxin, a small protein, represents another prevalent thiol-based redox enzyme system and plays important roles in protecting proteins from oxidative damage [18, 19]. For example, reduced thioredoxin, which is produced by thioredoxin reductase (encoded by *trx*), is involved in the repair of oxidized proteins through the cysteine thiol-disulfide exchange mechanism [20]. Consequently, the cellular concentration of NAD(P)H is critical, because this biomolecule serves as the main source of reducing power for cellular factors, such as thioredoxin, mycothiol, and peroxidases [10, 21].

Significant progress has been made in recent years on identifying the proteins that play regulatory roles in the oxidative-stress responses in *C. glutamicum*. Remarkably, numerous regulatory proteins participate in these responses. Moreover, many of these proteins contain cysteine residues in a configuration that may respond to cellular redox signals, thereby regulating cognate stress-responsive genes. These cysteine-containing regulators in *C. glutamicum* include WhcE and WhcA [22, 23], OxyR [8, 24], OhsR [25], RosR [26], MsrR [27], CosR [28], QorR [29], OasR [30], and OsrR [31]. In addition, along with the master regulator SigH [32, 33], multiple regulatory proteins also participate directly or indirectly in the regulation of oxidative-stress responses [34, 35].

Recently, Jeong et al. [36] have found that the *osnR* gene plays a negative role in the oxidative-stress responses in *C. glutamicum* and suggested a role for this gene in redox-mediated stress-response systems. Additionally, the *osnR*-overexpressing strain shows retarded growth, decreased transcription of the *trx* and *mtr* genes, and sensitivity to oxidants, such as H_2_O_2_ and diamide. However, the precise molecular function of *osnR* is still unclear. Accordingly, this study aimed to unveil the role of the *osnR* gene at the molecular level.

## Results

### Overexpression of the *osnR* gene affects the mycothiol metabolism

**Fig. 1:**
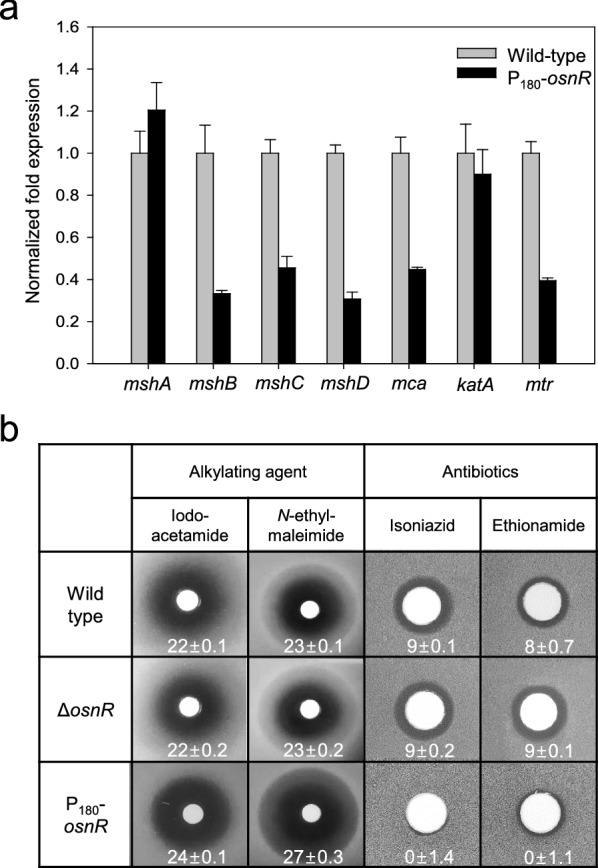
The mRNA levels of mycothiol biosynthetic genes in *C. glutamicum* cells and the sensitivities of the *C. glutamicum* mutants to alkylating agents and antibiotics. **a** *C. glutamicum* wild-type and *osnR*-overexpressing (P_180_-*osnR*) cells were grown in the minimal medium, and the mRNA levels were measured using qRT-PCR as described in “Materials and methods” section. Error bars indicate the standard deviation of three replicates from a representative experiment. **b** Paper discs placed on MB plates containing lawns of *C. glutamicum* wild-type, *osnR*-deleted (Δ*osnR*), or P_180_-*osnR* cells were spotted with iodoacetamide, *N*-ethylmaleimide, isoniazid, or ethionamide. Diameters of the zone of inhibition are shown in millimetres

Previously, we have reported that the *osnR* gene plays a negative regulatory role for the genes involved in reductant-dependent ROS detoxification [36]. Further, Jeong et al. [36] have found that the *osnR*-overexpressing strain (P_180_-*osnR*) shows an imbalanced NADPH/NADP^+^ ratio and downregulated transcription of genes encoding thioredoxin reductase (*trx*) and mycothiol disulfide reductase (*mtr*), and thus postulated that the strain is deficient in the redox metabolism. Since MSH is known as the major low-molecular-weight thiol, playing important roles in protecting *C. glutamicum* cells from oxidative stress, we analyzed the transcription levels of genes involved in mycothiol biosynthesis in P_180_-*osnR* strain. Genes, such as *mshB* (MSH deacetylase, cg1250), *mshC* (MSH ATP-dependent ligase, cg1709), and *mshD* (MSH acetyltransferase, cg2847) showed 55–70% decreased transcription compared with that of the wild type strain (Fig. 1a), suggesting a deficient mycothiol metabolism in P_180_-*osnR* cells. The *mca* gene (MSH *S*-conjugated amidase, cg1127), which is involved in the regeneration of mycothiol from the mycothiol-mediated detoxification product [17], also showed decreased transcription. Unlike these genes, *mshA* (MSH glycosyltransferase, cg0481), which encodes the first enzyme of the five-step biosynthesis process, showed marginal transcriptional upregulation in P_180_-*osnR* strain. Meanwhile, the transcription of the catalase-encoding *katA* gene, which was used as the positive control [36], was only marginally affected (Fig. 1a).

To test whether the altered transcription levels of mycothiol biosynthetic genes in P_180_-*osnR* strain decreased the cellular concentration of mycothiol, we compared the sensitivity of P_180_-*osnR* cells to alkylating agents with that of the wild-type cells because intracellular mycothiol is known to be involved in the detoxification of these compounds [13, 16]. As shown in Fig. 1b, P180-*osnR* cells showed an increased sensitivity to the thiol-attacking alkylating agents, such as iodoacetamide and *N*-ethylmaleimide, suggesting a diminished level of mycothiol in this strain. No significant difference in sensitivity was observed between the wild type and Δ*osnR* strains. Further, we tested the response of P_180_-*osnR* strain to antibiotics, such as isoniazid and ethionamide, which are pro-drugs that need to be activated by mycothiol [37, 38]. As expected, P_180_-*osnR* strain showed significant resistance to the antibiotics (Fig. 1b), consistent with a diminished level of intracellular mycothiol [12]. Collectively, these data show that the expression of the genes involved in the mycothiol metabolism might have been affected by the overexpression of *osnR*, whereby the cytosolic redox homeostasis involving mycothiol is disturbed.

### Identification of potential targets of OsnR via ChIP-seq

**Fig. 2:**
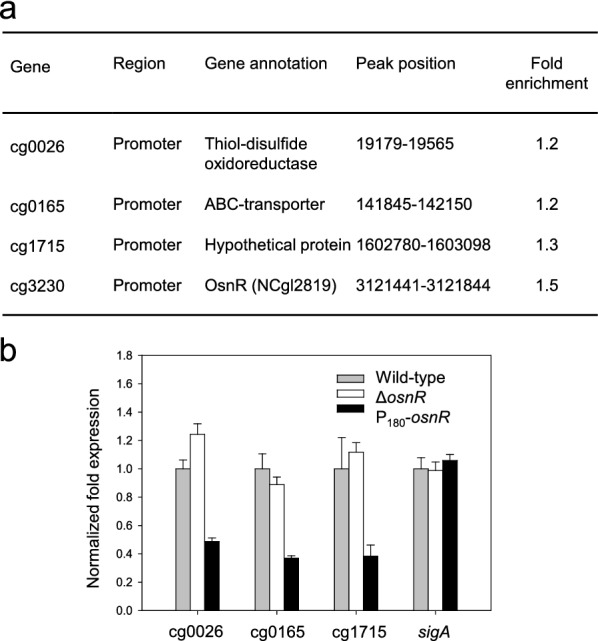
Target sites of the OsnR protein, as identified through the ChIP-seq analysis, and transcription levels of the identified genes in *C. glutamicum* cells. **a** The table shows the binding region of the Myc-tagged OsnR protein on the chromosomal DNA, as identified through the ChIP-seq analysis (see the “Materials and methods” section for the experimental details). **b** qRT-PCR analysis of the identified genes in *C. glutamicum* cells. The mRNA levels in cells grown in the minimal medium were measured. Three independent experiments were performed, and the data represent three technical replicates from a representative experiment

Because OsnR affected the transcription of various genes involved in redox reactions [36] and the mycothiol metabolism (Fig. 1a), we postulated that *osnR* has a general regulatory role in the redox metabolism. Therefore, we performed a ChIP-seq analysis using HL1653 strain, which over-expresses the Myc-tagged OsnR protein (Myc-OsnR), to identify direct chromosomal targets of the OsnR protein. Because specific antibodies against the OsnR were not commercially available, we used a Myc-tagged OsnR protein for the analysis. As shown in Fig. 2a, although only a few target sites were identified, all of them were located in the promoter or regulatory regions of genes, including cg0026, cg0165, cg0175, and cg3230 (*osnR*), suggesting a transcriptional regulatory function of the OsnR protein. Although the values of fold enrichment were rather low, the identified targets passed the threshold point, indicating that the values are statistically meaningful. Interestingly, the promoter region of *osnR* was identified as the most prominent target of the OsnR protein. The other targets included the promoter regions of cg0026 (thioredoxin domain-containing protein) and cg0165 (ABC-type transporter) genes. In particular, cg0026 encoded a protein that showed 42.8% identity to the DsbA-like thiol-disulfide oxidoreductase of *Streptomyces coelicolor*, which seems to function in the membrane-associated redox metabolism [39]. In accordance with the ChIP-seq data, the transcription of the identified genes cg0026, cg0165, and cg1715 was notably decreased in P_180_-*osnR* strain (Fig. 2b).

### OsnR directly binds to the promoter of *osnR*

**Fig. 3:**
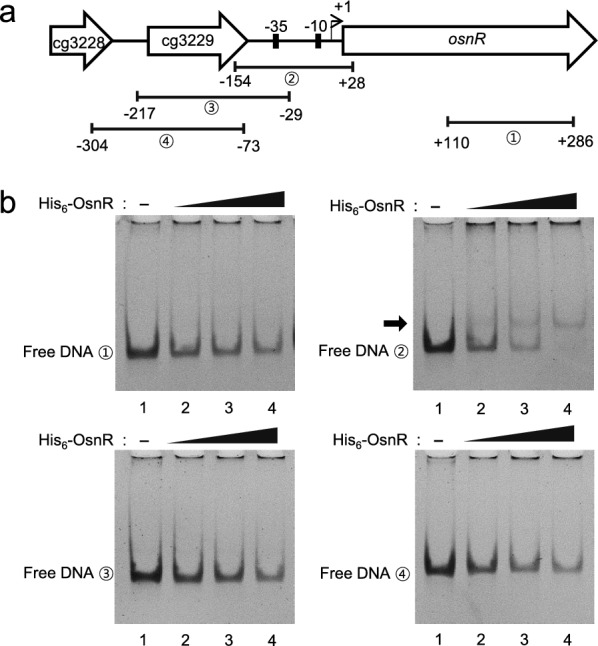
Binding of the purified OsnR protein to the promoter region of the *osnR* gene. **a** Schematic diagram of the chromosomal region comprising the *osnR* and adjacent genes. Numbers in circles indicate the DNA fragments used in the EMSA. **b** EMSA was performed using the purified His_6_-OsnR protein and the DNA fragments shown in **a**. The reactions in lanes 1, 2, 3, and 4 contained 0, 0.8, 1.0, and 1.2 μg of the OsnR protein, respectively. The arrow indicates the shifted band

Based on the ChIP-seq data, we assessed for DNA–protein interaction in a purified system. The overexpressed OsnR protein was purified in the form of histidine-tagged protein (His_6_-OsnR), which was obtained from inclusion bodies after the on-column refolding process. As shown in Fig. 3b, electrophoretic mobility shift assays (EMSA) showed binding of the His_6_-OsnR to the promoter region (from − 154 to + 28) of its own gene, as evidenced by the shifted bands, whereas DNA involving the upstream region of the promoter (from − 217 to − 29) did not, suggesting that the binding sites are located in the DNA region spanning from − 29 to + 28 (the transcriptional start site (+ 1) was based on published data [40]). The location of the presumed binding site is in accordance with the findings of Jeong et al. [36], who reported the negative regulatory role of the OsnR protein. As expected, DNA fragments containing either the promoter region of the upstream gene cg3229 or the coding region of the *osnR* gene (from + 110 to + 286) did not show any shifted band (Fig. 3b).

**Fig. 4:**
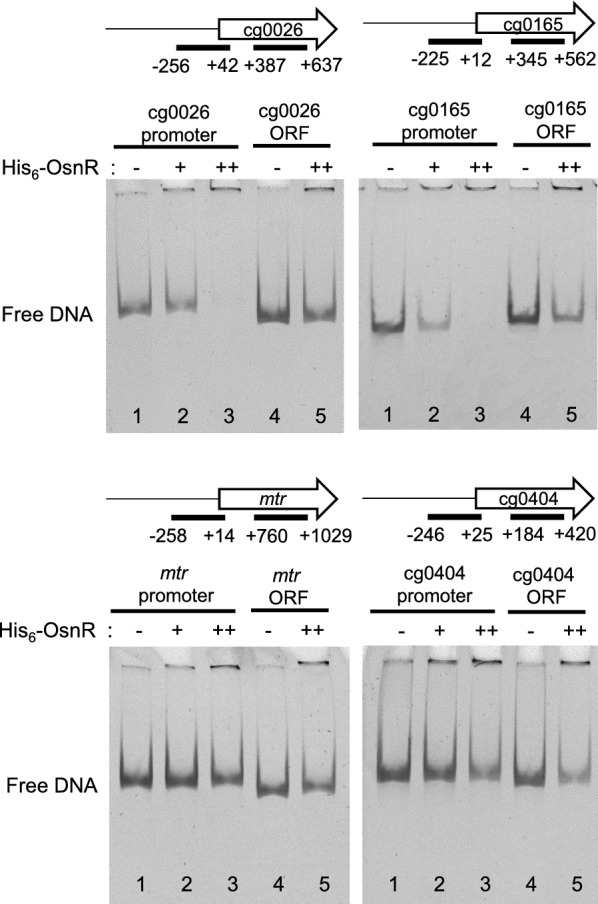
DNA binding of the purified His_6_-OsnR protein to the promoter regions of the putative target genes. The promoter and ORF regions are indicated with bars. Lanes 1 and 4 contained no protein, and lanes 2, 3, and 5 contained 0.6, 1.2, and 1.2 μg His_6_-OsnR, respectively

We further analyzed the binding of the OsnR protein on additional promoters identified in the ChIP-seq analysis. As shown in Fig. 4, although band shifts were not evident with the promoter regions of the cg0026 (from − 256 to + 42) and cg0165 (from − 225 to + 12) genes, free DNA clearly disappeared in the presence of His_6_-OsnR, suggesting a DNA–protein interaction. The disappearance of free DNA was not observed with DNA fragments containing the internal coding region of the genes. We also tested the binding of OsnR on the promoter region of additional genes, whose expression was affected by the overexpression of *osnR* [36]. No DNA-OsnR interaction was observed with the promoter region of the *mtr* (from − 258 to + 14) and cg0404 (a nitroreductase-family protein, from − 246 to + 25) genes [36]. In addition, OsnR was not observed to bind to the promoters of *trxB* and *sodA*, either (Additional file 1: Fig. S1). Overall, these data show that OsnR functions as a DNA-binding transcriptional factor, which may also repress its own transcription.

### The DNA-binding activity of OsnR is redox-dependent

**Fig. 5:**
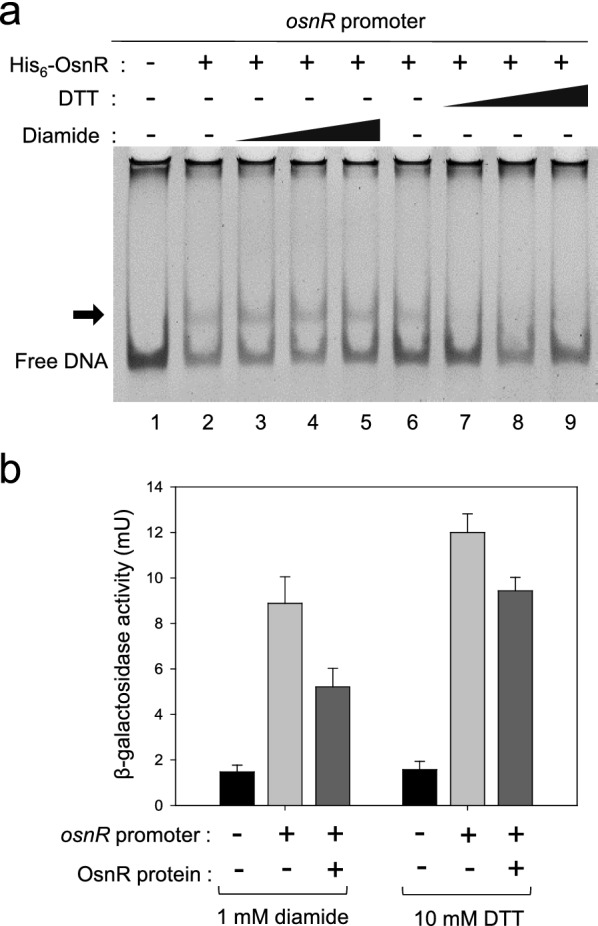
Effects of diamide and DTT on the DNA-binding activity of OsnR. **a** EMSA assay showing the effect of diamide (lanes 3–5) or DTT (lanes 7–9) on the DNA-binding activity of the OsnR protein. The binding of the purified His_6_-OsnR on the promoter region of *osnR* was assayed using EMSA. The experimental details are shown in the Material and Methods section. **b** An in vivo β-galactosidase assay showing the binding activity of OsnR on the promoter region of the *osnR* gene. The binding activity was assessed by measuring the β-galactosidase activity expressed from the P_*osnR*_::*lacZ* reporter plasmid. The *E. coli* host contained a plasmid that expresses the OsnR protein and the reporter. Cells were grown in the presence of DTT or diamide.The shown β-galactosidase activity is the mean value from at least three independently performed experiments

Based on the data that OsnR may play an autoregulatory role for the transcription of its own gene, we tested the effect of the redox status of the reaction mixture on the DNA-binding activity of the protein. As shown in Fig. 5a, the addition of the oxidizing agent diamide stimulated OsnR binding to the promoter region spanning from − 154 to + 28. However, when diamide was replaced with DTT, not only the stimulatory effect but also the shifted bands disappeared. This observation suggests that OsnR binds to the regulatory region of its own gene in cells exposed to oxidative stress, thereby inhibiting the transcription of the gene. To test whether the binding of OsnR represses its own transcription, we designed an in vivo experiment as described below. First, we constructed a reporter plasmid carrying the *osnR* promoter fused to the upstream region of the *lacZ* gene. Next, the constructed plasmid pSL553-P_*osnR*_::*lacZ* was introduced into *E. coli* cells along with plasmid pKK223-3-P_tac_::*osnR*. In the resulting strain, the OsnR protein could be induced with IPTG, and the binding of the expressed OsnR protein to the *osnR* regulatory region could be monitored by measuring the β-galactosidase activity. When cells carrying the reporter plasmid pSL553-P_*osnR*_::*lacZ* along with the empty vector pkk223-3 were grown with DTT, approximately 12 mU of β-galactosidase activity was observed, indicating that the *osnR* promoter is recognized by the *E. coli* transcription apparatus. The introduction of plasmid pKK223-3-P_tac_::*osnR* into the *E. coli* strain carrying the reporter plasmid and subsequent expression of the OsnR protein resulted in approximately 20% reduction in the β-galactosidase activity (Fig. 5b). Conversely, cells grown in the presence of diamide showed 40% reduction in the β-galactosidase activity. These data suggest that the binding of the OsnR protein to the regulatory region of the *osnR* gene is stimulated by diamide, whereby the expression of the *lacZ* gene is decreased. Collectively, these data suggest that OsnR functions as a transcriptional repressor and the DNA-binding activity of the OsnR protein is modulated by cellular redox status.

### Global transcriptional regulatory role of OsnR

**Fig. 6:**
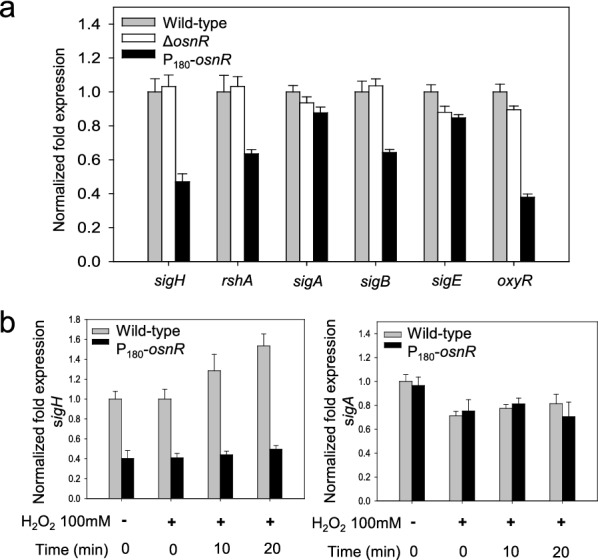
The transcription levels of σ-factor–encoding genes in *C. glutamicum* cells, and the effect of the hydrogen peroxide on the transcription of *sigH*. **a** *C. glutamicum* wild-type, *osnR*-deleted (Δ*osnR*), and *osnR*-overexpressing (P_180_-*osnR*) cells were grown in the minimal media, and the mRNA levels were measured using qRT-PCR. **b** The transcription levels of *sigH* and *sigA* in *C. glutamicum* wild-type and P_180_-*osnR* cells after challenging the cells with hydrogen peroxide. Error bars indicate the standard deviation of three replicates from a representative experiment

Because OsnR did not show direct binding to the promoter regions of the oxidative-stress-responsive genes *mtr* and *trx*, which showed decreased transcription in P_180_-*osnR* strain, we postulated that *osnR* may play an indirect regulatory role, conveying its activity through other regulatory genes. Moreover, the severe growth impairment of P_180_-*osnR* strain and the down-regulation of redox-responsive genes in the strain [36] also suggested a role of the *osnR* gene in a global regulatory cascade. Among the master regulatory genes, which might be involved in the regulation of stress-responsive genes, we chose the *sigH* gene (cg0876), which not only plays a major regulatory role in stress responses but is also involved in the transcription of *trx*, *mtr*, and mycothiol metabolic genes [33, 41, 42]. We also observed that the *sigH*-regulated *ftn* and *dps* genes, which are implicated in the iron homeostasis [43, 44], were down-regulated in P_180_-*osnR* strain (Additional file 1: Fig. S2). Based on this knowledge, we studied the transcription of *sigH* in the Δ*osnR* and *osnR*-overexpressing P_180_-*osnR* strains. As shown in Fig. 6a, the transcription level of the *sigH* gene in P_180_-*osnR* strain was at 50% of that observed in the wild type and Δ*osnR* strains. The gene *rshA*, which constitutes an operon with *sigH* [33, 41] and encodes an anti-sigma-factor, showed decreased transcription in P_180_-*osnR* strain. The *rshA* gene is also known to have its own promoter, which is σ^H^-dependent. Conversely, the expression level of *sigA*, encoding a housekeeping sigma factor, was only marginally affected in P_180_-*osnR* strain, whereas that of *sigB*, which encodes a primary-like sigma factor σ^B^ and plays roles in the transition phase between the exponential and stationary growth phases, was decreased by approximately 30% of that of the wild type strain (see below). These results suggest that transcriptional changes observed in many oxidative-stress response genes in P_180_-*osnR* strain might be in part due to decreased expression of *sigH,* which constitutes the large group of the σ^H^ regulon, of which many genes, including *sigB*, are members [42, 45]. Next, we measured the transcription level of the regulatory gene *oxyR* (cg2109), which acts as a transcriptional repressor for the *katA* gene. As shown in Fig. 6a, the *oxyR* gene was transcribed only at a 40% level in P_180_-*osnR* cells, compared with the wild-type level. This observation agrees with the findings of Jeong et al. [36], who reported the de-repression of *katA* in P_180_-*osnR* strains.

To elucidate the regulatory interaction between the *osnR* and *sigH* genes, we performed a physiological analysis as described below. First, *C. glutamicum* cells were grown to the mid-exponential growth phase in the minimal medium and were challenged with H_2_O_2_, which can oxidize protein thiols [46]. Next, the cells were harvested at appropriate time points, and the cellular *sigH* mRNA levels were quantitated via qRT-PCR. As shown in Fig. 6b, the H_2_O_2_ challenge caused increased transcription of *sigH* in wild-type cells. Stimulation of *sigH* was not observed in OsnR-overexpressing P_180_-*osnR* cells, and the level of transcription was at 40% of that of the wild-type cells. However, the transcription of *sigA* was almost identical in the wild-type and P_180_-*osnR* strains regardless of the H_2_O_2_ challenge. Collectively, these data suggest that the *osnR* gene may regulate the transcription of *sigH*.

**Fig. 7:**
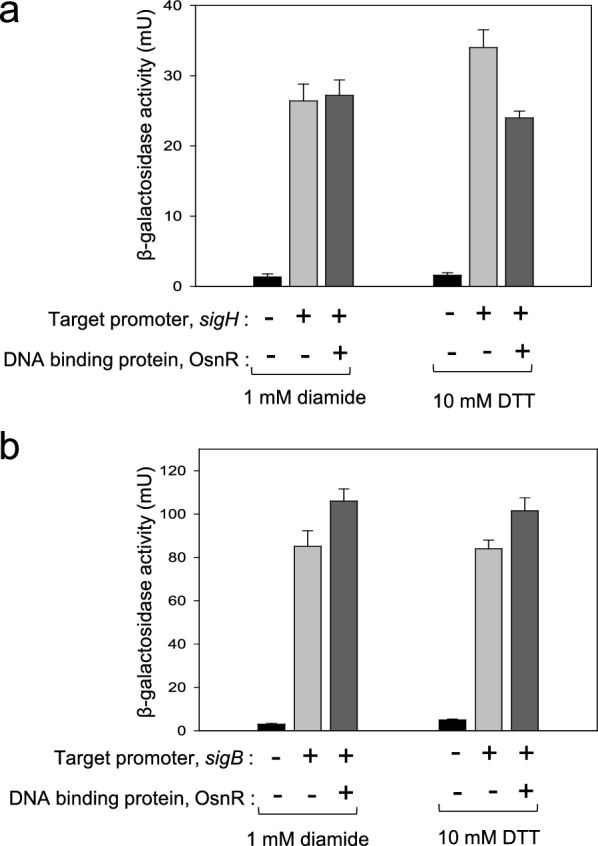
In vivo assays showing the binding of the OsnR protein to the promoter region of the *sigH* gene. The binding activity was analyzed by measuring the β-galactosidase activity expressed from the P_*sigH*_::*lacZ* reporter plasmid (**a**) and the P_*sigB*_::*lacZ* reporter plasmid (**b**). The *E. coli* host contained a plasmid that expresses the OsnR protein and the reporter. A plasmid carrying only the reporter was used as a negative control (**b**). The cells were grown with DTT or diamide. Three independent experiments were performed, and the data represent three technical replicates from a representative experiment

As the next step, we performed EMSA with the purified OsnR protein. As mentioned in the earlier section, the protein was purified in the form of His_6_-OsnR through the on-column refolding process. Although there were signs of protein binding on the regulatory region of *sigH*, it was not evident (see “Discussion”). Suspecting a weak interaction between the protein and target DNA, we switched to in vivo assays to quantitatively monitor the interaction. First, we constructed a reporter plasmid carrying the *sigH* promoter fused to the *lacZ* gene to monitor the *sigH* promoter activity through β-galactosidase activity. Next, the constructed plasmid pSL553-P_*sigH*_::*lacZ* was introduced into *E. coli* cells along with plasmid pKK223-3-P_tac_::*osnR*. Subsequently, the binding activity of the expressed OsnR protein to the *sigH* regulatory region was quantitated in cells grown with DTT or diamide. As shown in Fig. 7a, the presence of OsnR in cells grown in the presence of the reductant DTT repressed the β-galactosidase activity by 30%, suggesting that the OsnR protein bound to the regulatory region of the *sigH* gene. However, when cells were grown in the presence of diamide, the repression of the *sigH* gene was not observed. Next, the binding of the OsnR protein to the promoter region of the *sigB* gene, which also showed decreased transcription in P_180_-*osnR* cells, was tested through the constructed plasmid pSL553-P_*sigB*_::*lacZ*. In contrast to the findings regarding the *sigH* gene, no significant reduction of β-galactosidase activity was observed in cells grown with DTT or diamide (Fig. 7b), indicating that the interaction between the regulatory region of *sigH* and OsnR is specific. Collectively, these data suggest that cells exposed to oxidative stress initiate the transcription of the *sigH* gene by eliminating the repression exerted by the OsnR protein on the regulatory region of *sigH*.

## Discussion

The *osnR* gene has been suggested to play a global regulatory role as well as a negative role in redox-associated stress responses [36]. In this study, we demonstrated its DNA-binding activity as a transcriptional repressor for its own gene and cg0026, which is presumably involved in the redox-metabolism. The binding of the OsnR protein to the regulatory region of its own gene was evident, although weak interactions with other promoters were observed as well. Overexpression of the protein in *E. coli* and refolding process, which were applied to obtain pure OsnR protein, might be attributed to the low activity of the protein. Further, considering the small size of the protein (127 amino acids), histidine-tagging (His_6_-OsnR) may have hindered the interaction between OsnR and target DNA fragments. Nevertheless, weak binding of OsnR to the targets, such as *sigH* regulatory region, may be an intrinsic property of the interaction because such a property can be beneficial to cells, when one critically considers the inhibitory role of the *osnR* gene in cell physiology [36]. For instance, since the *sigH* gene is involved in the regulation of a broad spectrum of cellular activities, active regulation of *sigH* by a strong interaction with OsnR, whose role is confined to responses involving the redox homeostasis, may be physiologically inadequate to cells.

The binding of the purified OsnR on the regulatory region of its own gene shows that the *osnR* gene is autoregulated. Autoregulation of the *osnR* gene may suggest its critical role in cell physiology, because autorepression, known as one of the most efficient regulatory mechanisms [47], may save the response time of cells to changing environmental conditions. Similarly, the *sigH* gene is also known to be autoregulated by its own gene product [33, 48]. The OsnR binding site, located between − 29 and + 28 of the *osnR* gene, indicates that the OsnR protein functions as a repressor. Stimulation of the binding of OsnR to its own promoter in the presence of the oxidant diamide agrees well with previous reports, which showed a negative regulatory role of the gene [36]. Logically, cells exposed to diamide will downregulate OsnR, due to transcriptional repression of the *osnR* gene by its own protein, thereby de-repressing the stress-responsive genes. In addition, changes in cellular redox status can cause structural modification of the OsnR protein through cysteine residues, resulting in changes in the activity and functional properties of the protein, as such mechanism was proposed for other regulators, such as CosR (C49 and C62), MsrR (C62), OasR (C95), OhsR (C125), OxyR (C199 and C208), RosR (C92), and QorR (C17) [25–30, 49–51]. The OsnR protein has two cysteine residues (C2 and C10) at the N-terminus, and these residues may function as a thiol-based redox switch to respond to cellular redox status. Further, since the OsnR protein is predicted to form a homo-dimeric protein through the N-terminus, its dimerization capability could be modulated by cellular redox status.

Identification of only a handful of genes via the ChIP-seq analysis may suggest a global role of the *osnR* gene, which may exert its regulatory effect through other master regulators, such as *sigH*. However, as mentioned above, the intrinsically weak DNA–protein interaction might have resulted in unveiling only a few target genes. The growth condition employed for the assay may not be ideal for the OsnR protein to bind to target DNAs. In addition, the Myc-tagging of the OsnR protein may have lowered the binding activity of the protein, thus diminishing the number of identified target genes. The OsnR protein contains a helix-turn-helix DNA-binding motif at the C-terminus. Although the Myc-tag at the N-terminus worked well as an epitope for the binding of the 9E10 antibody, the tagging might have altered the protein conformation, thereby altering the dimerization capacity and, subsequently, the DNA-binding ability of the protein. Indeed, such negative effects have been reported in studies involving ChIP-seq [52–55].

Although we do not have a clear picture of how the *osnR* gene exerts its regulatory role in *C. glutamicum*, it is evident that it functions in the gene network involving the ECF-type sigma factor σ^H^, which primarily functions in heat- and oxidative-stress responses [22, 41]. The in vivo assays (Fig. 7) showed that the repression of *sigH* by OsnR is relieved in cells challenged with diamide, indicating a close regulatory interaction between *osnR* and *sigH*. The findings by Kim et al. [32], which showed regulated expression of the *sigH* gene, suggest the involvement of transcriptional factors, among which OsnR may be one of them. Likewise, the LexA repressor, which is involved in the regulation of the SOS response in *C. glutamicum*, also seems to participate in the regulation of the *sigH* gene, as suggested by the presence of a SOS box in the regulatory region of the *sigH* gene [33, 56].

The transcriptional downregulation of the mycothiol metabolic genes *mshB*, *mshC*, *mshD*, and *mca* in the *osnR*-overexpressing cells seems to be caused by the low intracellular level of σ^H^, because these genes have been reported to be part of the σ^H^ regulon [17, 33, 57, 58]. This conclusion is further supported by the fact that the *mshA* gene, which did not show notable transcriptional changes in P_180_-*osnR* cells, is not a member of the *sigH* regulon in *C. glutamicum* [59, 60]. The *sigH* gene is known to occupy the central position in the cross-regulated network of sigma factors in controlling genes involved in various stress responses, even the SOS response [29, 32, 33, 41, 57]. The general down-regulation of redox-responsive genes in P_180_-*osnR* strain could be due to a subordinate regulatory effect, as cross-regulation among sigma factors are frequent, and multiple sigma factors are often involved in the regulation of some genes [42, 61]. The general growth defect of P_180_-*osnR* strain [36] may support this idea. Demonstration of the OsnR binding to the regulatory region of *sigH* is necessary to demonstrate the role of OsnR in the transcription of the *sigH* gene.

The identification of cg0026 as one of the targets of OsnR in the ChIP-seq, and the binding of OsnR to the regulatory region of cg0026 (Fig. 4) is noteworthy. These observations indicate that the *osnR* gene specifically regulates genes involved in cellular redox reactions and globally participates in stress responses through *sigH*. The cg0026 gene has been annotated to encode a secreted protein containing a thioredoxin domain (i.e. thiol-disulfide oxidoreductase) and forms an operon with cg0025. The cg0025 gene encodes an integral membrane protein, which shows 27–33% identity with the cytochrome-biogenesis protein CcdA. Protein disulfide-isomerases, such as thiol-disulfide oxidoreductase, play important roles not only in the formation of disulfide bonds but also in reducing incorrect disulfide linkages [39]. Protein folding in the outer vicinity of the cell membrane is challenging due to the oxidative nature of the environment. In *Escherichia coli*, DsbA, which harbors a thioredoxin-like fold [62], catalyzes the formation of disulfide bonds in unfolded proteins as they are secreted into the periplasm. The DsbA active site involves a reactive disulfide bond found in a CXXC consensus sequence [62]. DsbA is regenerated by the membrane protein DsbB, which exchanges the disulfide bonds [63, 64]. Additionally, damaged periplasmic proteins by oxidative stress can be repaired by the periplasmic DsbC and membrane-bound DsbD protein pair [39]. Furthermore, CcdA, which is a functional homolog of DsbD, provides reducing equivalents for the reduction of cytochrome c. *Corynebacterium* species secrete cysteine-containing proteins into the exoplasm [65, 66]. In *C. diphtheria*, the membrane-localized disulfide-bond-forming reactions are catalyzed by MdbA and VKOR-like proteins, which are DsbAB-like proteins [39]. However, the proteins encoded by cg0026 and cg0025 do not show significant homology to the proteins, suggesting a distinctive role of the cg0026-encoded protein in *C. glutamicum*. Considering the role of *osnR* in responses involving oxidative stress, and the formation of a single transcriptional unit with cg0025, it is logical to speculate that the cg0026-encoded protein may play a role analogous to that of the DsbC of *E. coli*. Further studies are necessary to elucidate whether the cg0026 protein is involved in repairing non-native disulfide bonds in the exoplasm.

## Conclusions

We found that the *osnR* gene specifically regulates genes involved in redox-dependent stress responses. OsnR functions as a transcriptional repressor and responds to cellular redox status. The *osnR* gene may also participate in global transcriptional regulation through other regulators, such as *sigH*, which is known to play a master regulatory role in cells exposed to heat or oxidative stress. This work provides a deeper understanding of the redox-dependent stress-responsive regulatory networks of *C. glutamicum.*

## Materials and methods

### Bacterial strains, plasmids, and culture conditions

All the strains and plasmids used in this study are listed in Additional file 1: Table S1. *C. glutamicum* AS019E12 was used as the wild-type strain. *C. glutamicum* HL1638 and HL1643 were used as the Δ*osnR* mutant and *osnR*-overexpressing strains, respectively. *C. glutamicum* HL1653 harbours plasmid pSL580, which expresses the Myc-tagged OsnR protein (Myc-OsnR). *E. coli* DH10B (Invitrogen) was used to construct and propagate the plasmids. *E. coli* BL21 DE3 (Merck) was used for the expression of the His_6_-tagged OsnR protein (His_6_-OsnR). *E. coli* and *C. glutamicum* strains were cultured in Luria–Bertani (LB) broth at 37 °C and MB medium at 30 °C, respectively [67, 68]. MCGC minimal medium with 1% (wt/vol) glucose was prepared as described previously [36]. Antibiotics were added at the following concentrations: 50 μg/ml ampicillin, 25 μg/ml kanamycin, and 10 μg/ml tetracycline.

### Construction of plasmids

Standard molecular cloning and transformation methods were employed [68]. Plasmids were introduced into *C. glutamicum* cells via electroporation [67]. Restriction enzymes and DNA-modifying enzymes were used according to the manufacturer’s instructions (Takara Bio). PCR amplification of DNA from *C. glutamicum* AS019E12 chromosome was performed using the primers listed in Additional file 1: Table S2.

The plasmid expressing Myc-OsnR was constructed as follows: first, primers carrying the sequences needed for the amplification of the *osnR* gene were designed and additional sequences, which can optimally express the Myc epitope (5′-EQKLISEEDL-3′) in *C. glutamicum*. The resulting primers myc_*osnR*F and myc_*osnR*R (Additional file 1: Table S1) were used to amplify the chromosomal *osnR* gene. Following the amplification, the PCR product was digested with PstI and inserted into the PstI site of pSL360 [69]. The resulting plasmid pSL580, which expresses Myc-OsnR, was then introduced into *C. glutamicum* to generate strain HL1653. Over-transcription of the DNA encoding OsnR-Myc was verified by the RT-qPCR analysis (Additional file 1: Fig. S3). The pSL581plasmid expressing His_6_-OsnR was constructed by amplifying the *osnR* gene by using primers pET28a_*osnR*F and pET28a_*osnR*R, digesting the PCR product with NdeI and EcoRI, and subsequently inserting the resulting fragment in between the NdeI and EcoRI sites of pET28a vector (Novagen).

Plasmid pSL592, which can express the *osnR* gene in *E. coli*, was constructed by amplifying the *osnR* gene by using primers pKK223-3_*osnR*F and pKK223-3_*osnR*R, digesting the PCR product with EcoRI and PstI, and inserting the resulting fragment into pKK223-3 vector (Amersham Pharmacia). The reporter plasmid carrying *lacZ* was constructed as follows: first, the region of pRS415 [70] that contains the T1_4_, multiple cloning site, and *lac* operon was amplified using primers pRS415_F and pRS415_R. Subsequently, the PCR product was digested with ScaI, and the resulting fragment was inserted into pACYC184 vector (New England BioLabs), generating plasmid pSL553. Next, appropriate promoters were introduced into pSL553 as follows: the promoter and regulatory regions were amplified using primers pSL553_*osnR*F/pSL553_*osnR*R, pSL553_*sigH*F/pSL553_*sigH*R, and pSL553_*sigB*F/pSL553_*sigB*F, digested with SmaI, and inserted into pSL553 to generate plasmids pSL594 (P_*osnR*_::*lacZ*), pSL595 (P_*sigH*_::*lacZ*), and pSL596 (P_*sigB*_::*lacZ*), respectively. The orientations and identities of the inserts were verified via DNA sequencing (Macrogen, South Korea).

### RNA analysis

*Corynebacterium glutamicum* strains were grown in MCGC minimal media and harvested at the early stationary phase. When necessary, H_2_O_2_ was added to the cells during the mid-exponential growth phase to a final concentration of 100 mM, followed by 10–20 min incubation. After collecting the cells, their total RNA was extracted using the Nucleospin RNA II columns (Macherey–Nagel), and cDNA was synthesized using the ReverTra Ace qRT Kit (Toyobo). A CFX96 Real-Time PCR Detection System (Bio-Rad) was used as previously described [36, 71]. Reactions were performed in triplicate and relative ratios were normalized using the value for 16S rRNA. The primers used for qRT-PCR are listed in Additional file 1: Table S2.

### Physiological and biochemical analyses

Agar-diffusion assays were conducted as described previously [36, 71]. Lawn cells were mixed with 0.8% (v/v) top agar and poured onto MB plates. Paper disks (6.0 mm, Whatman), which were placed on the plates, were applied with the alkylating agent (10 μl of 100 mM iodoacetamide or *N*-ethlymaleimide) or antibiotic (200 mg isoniazid or ethionamide). The plates were photographed after 24 h of incubation at 30 °C.

Experiments involving measurement of β-galactosidase activity were performed as follows: *E. coli* cells carrying appropriate plasmids were grown in LB medium at 37 °C and treated with 0.2 mM IPTG at the OD_600_ of 0.5. The cells were then immediately treated with diamide or DTT and incubated at 30 °C for 3 h. Afterward, they were harvested, resuspended with the reaction buffer (5 mM Tris–HCl, 10 mM KCl, and 0.25% glycerol, pH7.5), and homogenized using the FastPrep-24 system (MP biomedicals). The supernatant obtained after centrifugation at 11,000×*g* for 5 min was used as the crude extract. The β-galactosidase assay was performed according to the report by Miller [72]. One unit of activity was defined as the amount of enzyme that hydrolyzed 1 μmol of ONPG in 1 min at 30 °C. The protein concentrations were determined using the Bradford assay with bovine serum albumin solutions as the standard solutions [73].

### ChIP-seq analysis

ChIP-seq analysis was performed by following the published methods [74] but with modifications. Myc-OsnR, constructed as described in the previous section, was used as bait to enrich the DNA segments with bound Myc-OsnR. Next, wild-type *C. glutamicum* cells or *C. glutamicum* HL1653 cells expressing Myc-OsnR were cultured in 100 ml MCGC medium at 30 °C to the final OD_600_ of ~ 2.0. Samples of 10 ml were collected and immediately mixed with formaldehyde (Extra Pure grade, Duksan) to give a final concentration of 1%, and incubated at 30 °C for 20 min with gentle agitation to induce DNA–protein cross-links. Subsequently, glycine was added to the final concentration of 125 mM, followed by incubation at room temperature for 5 min. The cells were harvested via centrifugation at 1600×*g* for 10 min and washed twice with ice-cold phosphate-buffered saline (pH 7.4). They were then resuspended in 0.5 ml lysis buffer composed of 1% sodium dodecyl sulfate, 1% Triton X-100, 10 mM EDTA, 50 mM Tris–HCl (pH 8.1), 1 mM PMSF, and 5 μg/ml RNase A, incubated at 30 °C for 10 min, and then chilled on ice. The lysate was subjected to sonication (microprobe diameter of 3 mm; Sonics & Materials, Inc.) for 20 s at 30% amplitude on ice. The sonication process was repeated 10 times with 30 s interval to obtain chromosomal DNA fragments of 200–500 bp. Cell debris was removed via centrifugation at 11,000×*g* for 10 min. The supernatant (5 μl) was collected and stored at − 80 °C for later use as the control input DNA. The rest of the cell extract was subjected to immunoprecipitation by using the Pierce Agarose ChIP kit (26156, Thermo Fisher Scientific). To immunoprecipitate the Myc-OsnR-bound DNA, 7 μg of c-Myc monoclonal antibody (9E10, Thermo Fisher Scientific) was added to the extract, and the mixture was incubated overnight at 4 °C. Then, 20 μl protein A/G and agarose beads (Thermo Fisher Scientific) were added to the mixture, followed by incubation at 4 °C for 2 h. The beads were washed twice with the wash buffer and resuspended with 150 μl elution buffer. The mixture was incubated at 65 °C for 30 min with shaking. The immunoprecipitated and control input DNA samples were diluted with the elution buffer and then treated with 20 μl proteinase K solution for 2 h at 65 °C. The samples were then purified using DNA clean-up columns (Thermo Fisher Scientific), precipitated, and resuspended in water. The resulting DNA samples were sequenced by Macrogen (South Korea) by using the HiSeq 4000 Sequencing System. All sequencing data have been deposited in ArrayExpress (accession number E-MTAB-11048).

### Purification of His_6_-OsnR and EMSA

*Escherichia coli* BL21 (DE3) cells (Merck Bio-science) carrying pSL581, which overexpress His_6_-OsnR, were cultivated in LB medium. Recombinant-protein expression was induced by the addition of 0.2 mM IPTG at the OD_600_ of 0.4. After cultivation for 4 h at 30 °C, the cells were harvested via centrifugation at 6000×*g* for 10 min, resuspended in 10 ml buffer (20 mM HEPES, 500 mM NaCl, 40 mM imidazole, 8 M urea, 0.5% Tween 20, and 5% glycerol, pH 7.4), and lysed via sonication. The lysate was centrifuged at 11,000×*g* for 1 h to remove cell debris. The resulting supernatant was filtered through a 0.2 μm syringe filter (Sartorius Stedim) before loading to a HisTrap FF column (GE Healthcare). Refolding of His_6_-OsnR was induced by applying 20 ml refolding buffer (20 mM HEPES, 500 mM NaCl, 40 mM imidazole, 0.5% Tween 20, 5% glycerol, and 5 mM DTT, pH 7.4) to the column in a linear gradient (0.1 ml/min). The proteins were then eluted using 5 ml elution buffer (20 mM HEPES, 500 mM NaCl, 500 mM imidazole, 0.5% Tween 20, and 5% glycerol, pH 7.4) and concentrated via ultrafiltration (Amicon, Millipore).

For EMSA, the purified His_6_-OsnR proteins (maximum of 1.2 μg) were incubated with 30 ng of DNA fragments, which were prepared using PCR with *C. glutamicum* genomic DNA as the template. The primers used for the PCR amplification are listed in Additional file 1: Table S2, and the resulting amplified DNA were 200–300 bp. The DNA–protein binding reaction was performed at 30 °C for 30 min in a total volume of 20 μl [20 mM HEPES, 2.5 mM MgCl_2_, 75 mM KCl, 0.5 mM EDTA, 2 mM DTT, 10% glycerol, 1 μg poly d(I-C), and 100 ng/μl BSA, pH 7.4]. When needed, diamide at the final concentration of 5, 10, or 20 mM, or DTT at 10, 20, or 40 mM were spiked into the protein sample. Bands were resolved via 5% polyacrylamide-gel electrophoresis, and DNA was visualized using GelRed nucleic acid stain (Biotium).

## Supplementary Information

**Additional file 1: Table S1.** Bacterial strains and plasmids used in this study. **Table S2.** Oligonucleotides used in this study. **Figure S1.** Binding of the purified OsnR protein on the promoter regions of the *trxB* and *sodA* genes. **Figure S2.** The mRNA levels of genes linked to iron homeostasis in *C. glutamicum* cells. **Figure S3.** Transcription of the *myc*-*osnR* fusion gene as measured by qRT-PCR. *C. glutamicum* cells were grown in minimal media.

## Data Availability

All data generated or analyzed during this study are included in this published article and its Additional files.

## Abbreviations

*C. glutamicum*: *Corynebacterium glutamicum*
ChIP-seq: Chromatin immunoprecipitation-sequencing
ROS: Reactive oxygen species
MSH: Mycothiol
EMSA: Electrophoretic mobility shift assay
*E. coli*: *Escherichia coli*
DTT: Dithiothreitol
IPTG: Isopropyl β-d-1-thiogalactopyranoside
ONPG: *O*-Nitrophenyl-β-d-galactopyranoside
ECF: Extracytoplasmic function
LB: Luria–Bertani
RT-qPCR: Reverse transcription-quantitative polymerase chain reaction
