# Supplementary material for: OsnR is an autoregulatory negative transcription factor controlling redox-dependent stress responses in Corynebacterium glutamicum
Source: Microb Cell Fact. 2021 Oct 18;20:203. doi: 10.1186/s12934-021-01693-1 (PMC8524982; doi:10.1186/s12934-021-01693-1)
Supplement: Supplementary file 1 — Additional file 1: Table S1. Bacterial strains and plasmids used in this study. Table S2. Oligonucleotides used in this study. Figure S1. Binding of the purified OsnR protein on the promoter regions of the trxB and sodA genes. Figure S2. The mRNA levels of genes linked to iron homeostasis in C. glutamicum cells. Figure S3. Transcription of the myc-osnR fusion gene as measured by qRT-PCR. C. glutamicum cells were grown in minimal media. [file 12934_2021_1693_MOESM1_ESM.docx]

**Table S1.** Bacterial strains and plasmids used in this study

| **Strain/plasmid** | **Relevant characteristics** | **Reference/source** |
| --- | --- | --- |
| **Strains** |  |  |
| *E. coli* |  |  |
| DH10B | F^‾^*mcr*A Δ(*mrr*-*hsd*RMS-mcrBC) *φ*80*lac*ZΔM15 Δ*lac*X74 *rec*A1 *end*A1 *ara*D139Δ(*ara, leu*)7697 *gal*U *gal*K *λ*-*rps*L *nup*G | [1] |
| ET12567 | *dam dcm hsd,* restriction-deficient | [2] |
| BL21DE3 | F^-^ *ompT hsdSB*(r_B_ ^-^ m_B_ ^-^) *gal dcm* (DE3) | Merck |
| HL1669 | BL21DE3 harboring pSL581, Km^R^ | This study |
| HL1727 | DH10B harboring pKK223-3 and pSL553, Ap^R^, Tet^R^ | This study |
| HL1728 | DH10B harboring pKK223-3 and pSL594, Ap^R^, Tet^R^ | This study |
| HL1729 | DH10B harboring pKK223-3 and pSL595, Ap^R^, Tet^R^ | This study |
| HL1730 | DH10B harboring pSL592 and pSL594, Ap^R^, Tet^R^ | This study |
| HL1731 | DH10B harboring pSL592 and pSL595, Ap^R^, Tet^R^ | This study |
| *C. glutamicum* |  |  |
| AS019 | Spontaneous rifampicin-resistant mutant of *C. glutamicum* ATCC13059 | [3] |
| AS019E12 | Restriction-deficient mutant of AS019 | [4] |
| HL1638 | *C. glutamicum* AS019E12 derivative, Δ*osnR* | [5] |
| HL1643 | *C. glutamicum* AS019E12 carrying pSL579, Km^R^ | [5] |
| HL1653 | *C. glutamicum* AS019E12 carrying pSL580, Km^R^ | This study |
| **Plasmids** |  |  |
| pSL360 | Overexpression vector pSK1CAT carrying P_180,_ Km^R^ | [6] |
| pSL579 | Overexpression vector carrying P_180_-*osnR* | [5] |
| pSL580 | Overexpression vector carrying P_180_-myc-*osnR* | This study |
| pET28a | N-terminal His_6_ tag expression vector, Km^R^, IPTG inducible | Merck |
| pSL581 | His_6_-tagged OsnR expression vector, Km^R,^ IPTG inducible | This study |
| pKK223-3 | Expression vector, Ap^R^, P*_tac_* | Amersham  Pharmacia |
| pSL592 | pKK223-3 carrying *osnR* | This study |
| pRS415 | Promoter-less *lac* operon fusion vector | [7] |
| pACYA184 | Low copy-number cloning vector carrying p15A replication origin;  Cm^r^, Tc^r^ | New England BioLabs |
| pSL553 | pACYC184 carrying pRS415[T14+MCS+lac operon], Tet^R^ | This study |
| pSL594 | pSL553 carrying *osnR* promoter | This study |
| pSL595 | pSL553 carrying *sigH* promoter | This study |
| pSL596 | pSL553 carrying *sigB* promoter | This study |

*ATCC, American Type Culture Collection.

**Table S2.** Oligonucleotides used in this study

| **Oligonucleotides** | **Sequence (5′→3′)** |
| --- | --- |
| For construction of the plasmid expressing Myc-fused OsnR | |
| myc_*osnR*F | CTGCAGTGACCATTGATAGATGGAACAGAAACTGATTAGCGAAGAAGACCTG  ATGTGTCAAAACATATATTTGG (*Pst*I site underlined) |
| myc_*osnR*R | CTGCAGGTTATTTATTAAGGGTTG (*Pst*I site underlined) |
| For construction of the plasmid expressing His_6_-fused OsnR | |
| pET28a_­*osnR*F | GGGAATTCCATATGATGTGTCAAAAC (*Nde*I site underlined) |
| pET28a_­*osnR*R | CGGAATTCAAGGGTTGGATGCTATGCG (*Eco*RI site underlined) |
| For construction of the Lac operon fusion plasmid | |
| pRS415_F | GCCACTCATCGCAGTACT AATAGGCGTATCACGAGGCCC (*Sca*I site underlined) |
| pRS415_R | ATGAATTACAACAGTACT TGGTGTGTGGGTTAGGTCTGG (*Sca*I site underlined) |
| pKK223-3_*osnR*F | GAATTCGGAAAGCCAAACAGTGACCA (*Eco*RI site underlined) |
| pKK223-3_*osnR*R | CTGCAG ATTAAGGGTTGGATGCTATG (*Pst*I site underlined) |
| pSL553_ *osnR*F | GGGATCGGAATTCCCGGGCACTAGAAATTCAG (*Sma*I site underlined) |
| pSL553_ *osnR*R | GTTGTCCGGATCCCCGGGGTGGAAAAAGTGGGATTGGC (*Sma*I site underlined) |
| pSL553_ *sigH*F | GGGATCGGAATTCCCGGGATCATCGTCATCTTTGGG (*Sma*I site underlined) |
| pSL553_ *sigH*R | GTTGTCCGGATCCCCGGGCATAATAAACCACCATCC (*Sma*I site underlined) |
| pSL553_ *sigB*F | GGGATCGGAATTCCCGGGCAGTCAATCCTAAGAGCTTG (*Sma*I site underlined) |
| pSL553_ *sigB*R | GTTGTCCGGATCCCCGGGCCTCCTGGATAAGATCCAG (*Sma*I site underlined) |
| qRT-PCR | |
| RT_16S_F | ACCCTTGTCTTATGTTGCCAG |
| RT_16S_R | TGTACCGACCATTGTAGCATG |
| RT_*mshA*_F | CAGGAGGAGATGCAGGATTTG |
| RT_*mshA*_R | GCGATCATTTCCAGGGCTATAA |
| RT_*mshB*_F | GGGTTTAGGATCCATGAGCTTTA |
| RT_*mshB*_R | TGCAGGATCACCAACCATAC |
| RT_*mshC*_F | TGTCGATGACCCTCTGTTTG |
| RT_*mshC*_R | ATGCTCAGGGCTTCCATATC |
| RT_*mshD*_F | GGACTTGGTCACTCCCATTT |
| RT_*mshD*_R | AGTTCCGTGGTTTCCTCATC |
| RT_*mca*_F | GAGCACAGATGGCTAGGTTATG |
| RT_*mca*_R | GGGTGACTTTATCCGAGTCTTC |
| RT_*katA*_F | GGACGTATCCGAATACACCAAG |
| RT_*katA*_R | CTCACCAGCAACAGTAGAGAAG |
| RT_*mtr*_F | CCCTTACATCCCTGAAGCTATT |
| RT_*mtr*_R | GCCACCAACAATCACCAAAG |
| RT_cg0026_F | GCAAAGTGGAGCGATGAAAC |
| RT_cg0026_R | GCTCAGATGGTTCTCCAAAGA |
| RT_cg0165_F | GTGGTAGGCCTTCTGTTGTATG |
| RT_cg0165_R | CCCAGAGAGTGGCGTAAATG |
| RT_cg1715_F | GTTCGACTACGCCACATGAT |
| RT_cg1715_R | GTAGTGCGCGAGTTCTTTCT |
| RT_*sigA*_F | CAACCTTCGTCTGGTTGTCT |
| RT_*sigA*_R | GAACTTGTAGCCCTTGGAGTAG |
| RT_*sigH*_F | GAATCAACTCAGCCCGGAATA |
| RT_*sigH*_R | GAGTGGAACGTCCATGATCTC |
| RT_*rshA*_F | ATCGGAGTTTCGTAGTCTGTTG |
| RT_*rshA*_R | TATTCCAGATGATGCTCGACAC |
| RT_*sigB*_F | TGCCACTGCTGGATCTTATC |
| RT_*sigB*_R | GTTGCGTAGGTGGAGAACTTA |
| RT_*sigE*_F | TAGTCGCAGAACATGCAGATAG |
| RT_*sigE*_R | TGTTTCTTGGGTCAGGTCTTC |
| RT_*oxyR*_F | CCGCCAAGACTTAGAACTATCC |
| RT_*oxyR*_R | CAGCAGTAGTGGAGCTAATGG |
| RT_*ftn*_F | AGGCACAGAGCAAAGAAGAG |
| RT_*ftn*_R | GATAGCGGAGGTGACATCAAG |
| RT_*dps*_F | CACCGACTACAACGATCTTCAC |
| RT_*dps*_R | GTCAGCATAGCCACGAACAA |
| EMSA | |
| EM_*osnR*(ORF)_F1 | CAAGATTACGAGGACGCTTCCG |
| EM_*osnR*(ORF)_R1 | GGCGAGGCCACGTTCAATCTTG |
| EM_*osnR*_F2 | ATTGTCTTGCTACTCGGCGGATA |
| EM_*osnR*_R2 | GCCAATTCGCGAGACAAGTAAAAG |
| EM_*osnR*_F3 | CCCTCAACGCTGGAGAAAACTCA |
| EM_*osnR*_R3 | CTATCAATGGTCACTGTTTGGC |
| EM_*osnR*_F4 | GCTGGGCCAGGGCCTTTACGAA |
| EM_*osnR*_R4 | CGGGCTCGCTTAATTTCAG |
| EM_*c*g0026_F | CAAGCGTGGGGATTCCTGGCTC |
| EM_*c*g0026_R | ACCAGTGTCGGTACGAGCCA |
| EM_*c*g0026(ORF)_F | CATCTTTGGAGAACCATCTGAGC |
| EM_*c*g0026(ORF)_R | GAAAGCTGGGGTGGAGTAGGCG |
| EM_*c*g0165_F | TTTGGTGGTGGGCAATGCTGCGA |
| EM_*c*g0165_R | CAGTGCCGGCATTGGTTTGACG |
| EM_*c*g0165(ORF)_F | GGCAGGCGCATTGGTGTCATCAG |
| EM_*c*g0165(ORF)_R | AAGTCCAGCGAATCAGCCTGC |
| EM_*mtr*_F | ATCATCGACAACGCTCGCCACGA |
| EM_*mtr*_R | GGCTGCTCAGACATAAGATG |
| EM_*mtr*(ORF) _F | GTGAAGATCTCCACCGACACC |
| EM_*mtr*(ORF)_R | CTGCAGGTCATTAGGGTTGGC |
| EM_*c*g0404_F | ACCGCTTCTAAGCACCCAACTC |
| EM_*c*g0404_R | CGCCTCGACGACTGAAAGTG |
| EM_*c*g0404(ORF)_F | CAAAATAGCCCCAATCGTCACA |
| EM_*c*g0404(ORF)_R | GTTGGAAACAGAGTCATGGTAG |
|  |  |


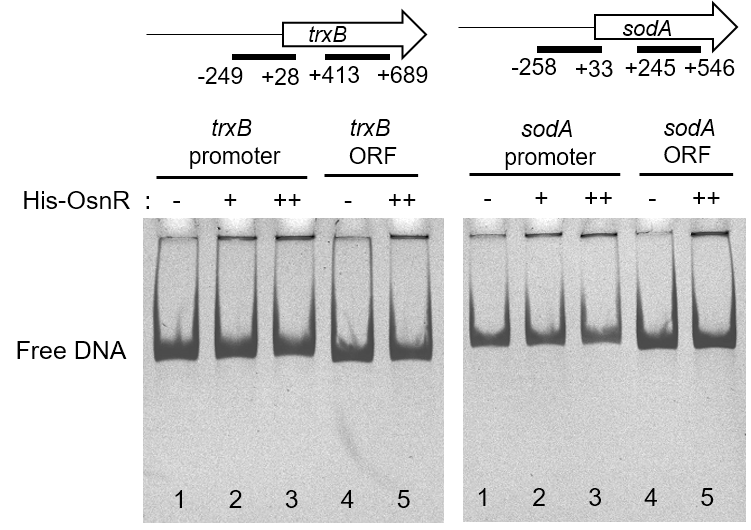


Fig S1. Binding of the purified OsnR protein on the promoter regions of the *trxB* and *sodA* genes. The promoter and ORF regions are indicated with bars. Lanes 1 and 4 contained no protein, while lanes 2, 3, and 5 contained 0.6, 1.2, and 1.2 μg His_6_-OsnR, respectively.


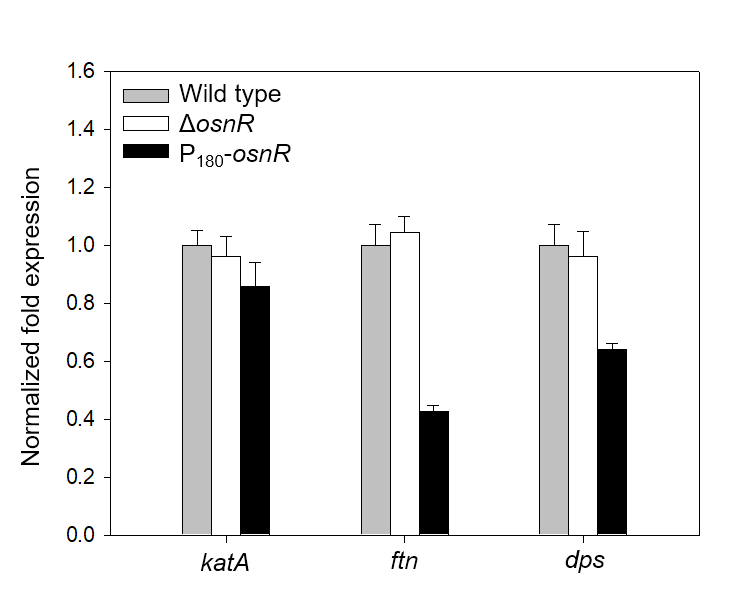


Fig S2. The mRNA levels of genes linked to iron homeostasis in *C. glutamicum* cells. *C. glutamicum* wild-type, *osnR*-deleted (Δ*osnR*), and *osnR*-overexpressing (P_180_-*osnR*) cells were grown in minimal media and the mRNA levels of the genes indicated were measured using qRT-PCR.

Fig. S3. Transcription of the *myc*-*osnR* fusion gene as measured by qRT-PCR. *C. glutamicum* cells were grown in minimal media. Bars indicate the levels of transcription in wild-type and *myc-osnR*-overexpressing (P_180_-*myc*-*osnR*) strains. The value for the wild-type strain was arbitrarily set to 1. The data represent one of the three independent experiments. Error bars indicate standard deviation of three replicates from a representative experiment.

**Supplementary Reference**

1. Grant SG, Jessee J, Bloom FR, Hanahan D. Differential plasmid rescue from transgenic mouse DNAs into Escherichia coli methylation-restriction mutants. Proc Natl Acad Sci USA. 1990;87:4645-9.

2. MacNeil DJ, Gewain KM, Ruby CL, Dezeny G, Gibbons PH, MacNeil T. Analysis of *Streptomyces avermitilis* genes required for avermectin biosynthesis utilizing a novel integration vector. Gene. 1992;111:61-8.

3. Yoshihama M, Higashiro K, Rao EA, Akedo M, Shanabruch WG, Follettie MT, Walker GC, Sinskey AJ. Cloning vector system for *Corynebacterium glutamicum*. J Bacteriol. 1985;162:591-7.

4. Follettie MT, Peoples OP, Agoropoulou C, Sinskey AJ. Gene structure and expression of the *Corynebacterium glutamicum* N13 *ask-asd* operon. J Bacteriol. 1993;175:4096-103.

5. Jeong H, Kim Y, Lee HS. The *osnR* gene of *Corynebacterium glutamicum* plays a negative regulatory role in oxidative stress responses. J Ind Microbiol Biotechnol. 2019;46:241-8.

6. Park SD, Lee SN, Park IH, Choi JS, Jeong WK, Kim Y, Lee HS. Isolation and characterization of transcriptional elements from *Corynebacterium glutamicum*. J Microbiol Biotechnol. 2004;14:789-95.

7. Simon RV, Houman F, Kleckner N. Improved single and multicopy *lac*-based cloning vectors for protein and operon fusions. Gene. 1987;53:85-96.
